# Supplementary material for: Evaluation of Sierra Leone’s Elimination of Mother-to-Child Transmission of HIV program, 2024: The need for a Life Stages approach to triple elimination
Source: PLOS Glob Public Health. 2026 Apr 28;6(4):e0005491. doi: 10.1371/journal.pgph.0005491 (PMC13123933; doi:10.1371/journal.pgph.0005491)
Supplement: S1 Table — (DOCX) [file pgph.0005491.s001.docx]

**Supplementary table 1:** **Rapid EMTCT Cascade Analysis Tool and Data Sources of health facilities, June 2024, Sierra Leone.**

| Name of data collector(s): |  |
| --- | --- |
| Date (D/M/Y): |  |
| Time: |  |
| Name of Health Facility: |  |
| Type of Health Facility: |  |
| Location (District, chiefdom): |  |
| Health Facility Catchment Area Population: |  |
| % Pregnant: |  |
| Time Period: |  |
| Expected No. of Pregnant Women: |  |
| Total number of pregnant women attended ANC for the period |  |

|  |  | **Total** | **Data Sources** |
| --- | --- | --- | --- |
| **Step 1** | **No. of clients with 1st ANC Visits** |  | Mother and Neonate register; HF3 form |
|  |  | | |
| **Step 2a** | **HIV Test at 1st ANC Visit (The total should be the number of 1^st^ ANC visits)** |  | Mother and neonate register, PMTCT register |
|  | - Previously identified HIV + at ANC1 |  | PMTCT register |
|  | - Tested: HIV + women |  | PMTCT register |
|  | - Tested: HIV - women |  | PMTCT register |
|  | - Not tested and not previously diagnosed (opted out) |  | PMTCT register |
|  | - Missing (not recorded) |  | Mother and neonate register |
|  |  | | |
| **Step 2b** | Syphilis Testing | | |
|  | - Total no. of pregnant women tested for syphilis at 1st ANC visit |  | Mother and neonate register |
|  | - Tested non-reactive (NR) for syphilis |  | Mother and neonate register |
|  | - Tested reactive (R) for syphilis |  | Mother and neonate register |
|  | - No. treated for syphilis if tested reactive (R) |  | Mother and neonate register |
|  |  | | |
|  | ART Cascade at ANC1 | | |
| **Step 3** | - No. of HIV + women newly diagnosed and initiated on ART at ANC1 |  | PMTCT register; ART Register |
|  | - No. of known HIV + women already on ART before ANC1(own health) |  | PMTCT Register; ART Register |
|  | **Total no. of HIV + women on ART at ANC1 during the reporting period (Jan-June 2021)** |  | PMTCT Register; ART Register |
|  | **No. of HIV + women who started ART < 4 weeks before delivery** |  | PMTCT Register |
|  |  |  |  |
| **Step 4** | **No. of HIV + women delivered in the health facility** |  | Labour and delivery register; ART care card (mother) |
|  | **No. of HIV + women who completed 4 ANC visits** |  | Mother and Neonate register; ART care card (mother) |
|  | **No. of HIV + women who completed 8 ANC visits** |  | Mother and Neonate register; ART care card (mother) |
|  | **No. of HIV + women who attended postnatal care follow up visit** |  | PNC register; PMTCT register; PMTCT follow up; ART care card (mother) |
|  | HIV Exposed Infant Cascade | | |
| **Step 5** | **No. of HIV exposed infants born to the HIV + women** |  | Exposed infant register; EID card; PMTCT follow up register; ART care card (mother) |
|  | **No. of HIV exposed infants delivered in a facility** |  | Exposed infant register |
|  |  |  |  |
| **Step 6** | **No. of HIV exposed infants initiated on ARV prophylaxis within 72 hours of birth** |  | Exposed infant register; EID card; PMTCT follow up register; ART care card (mother) |
|  | **No. of HIV exposed infants initiated on ARV prophylaxis after 72 hours of birth** |  | Exposed infant register |
|  |  | | |
| **Step 7** | **No. of HIV exposed infants initiated on cotrimoxazole less than 2 months after birth** |  | Exposed infant register; EID card; PMTCT follow up register; ART care card (mother) |
|  | **No. of HIV exposed infants initiated on cotrimoxazole two or more months after birth** |  | Exposed infant register |
|  |  | | |
| **Step 8** | **No. of HIV exposed infants with 1^st^ virological test (DNA PCR) done within 6-8 weeks of birth** |  | Exposed infant register; EID card; PMTCT follow up register; |
|  | - No. of HIV exposed infants with 6–8-week virological test result received |  | Exposed infant register; EID card; PMTCT follow up register; |
|  | - Infants identified as HIV + (reactive virological test result) |  | Exposed infant register; EID card; PMTCT follow up register; ART care card; ART register (adult and paediatrics) |
|  | - Eligible infants (HIV +) linked to ART care |  | Exposed infant register; EID card; PMTCT follow up register; ART care card; ART register (adult and paediatrics) |
|  |  | | |
| **Step 9** | **No. of HIV exposed infants with 2^nd^ virological test (DNA PCR) done 12 weeks after cessation of breastfeeding or as indicated** |  | Exposed infant register; EID card; PMTCT follow up register |
|  | - No. of HIV exposed infants with 2^nd^ virological test result received |  | Exposed infant register; EID card; PMTCT follow up register |
|  | - Infants identified as HIV + |  | Exposed infant register; EID card; PMTCT follow up register; ART care card; ART register (adult and paediatrics) |
|  | - Eligible infants (HIV +) linked to ART care |  | Exposed infant register; EID card; PMTCT follow up register; ART care card; ART register (adult and paediatrics) |
|  |  | | |
| **Step 10** | **No. of HIV exposed infants with HIV Rapid Antibody Test done at 18 months or thereafter** |  | Exposed infant register; EID card; PMTCT follow up register |
|  | - Children identified as HIV + |  | Exposed infant register; EID card; PMTCT follow up register; ART care card; ART register (adult and paediatrics) |
|  | - Eligible children (HIV +) linked to ART care |  | Exposed infant register; EID card; PMTCT follow up register; ART care card; ART register (adult and paediatrics) |
|  | - Children tested HIV negative, no longer breastfeeding |  | Exposed infant register; EID card; PMTCT follow up register |
|  | - Children with HIV status unknown |  | Exposed infant register |
|  |  |  |  |
| **Step 11** | Retesting Performance | | |
|  | Number of women tested for HIV after ANC 1 |  | Mother and Neonate register, PMTCT register |
|  | Number of HIV positive tests after ANC 1 |  | Mother and Neonate register, PMTCT register |
|  | Number of women tested for HIV during labour and delivery |  | Labour and delivery register; |
|  | Number of HIV positive tests during labour and delivery |  | Labour and delivery register; ART care card; ART register |
|  | Number of women tested for HIV during Postnatal care (6 weeks after delivery) |  | PNC Register; ART care card; ART register |
|  | Number of HIV positive tests during Postnatal care (6 weeks after delivery) |  | PNC Register; ART care card; ART register |
|  |  |  |  |

*ANC-Antenatal Clinic, ART-antiretroviral therapy, DNA-Deoxyribonucleic acid, eMTCT-elimination of mother-to-child transmission, EID-Early Infant Diagnosis, HF3: Health Facility Form 3, HIV: Human immunodeficiency Virus, HBV-Hepatitis B Virus, PMTCT-Prevention of Mother-to-Child Transmission, PNC-Post-natal clinic register*
