# Supplementary material for: Evaluation of Sierra Leone’s Elimination of Mother-to-Child Transmission of HIV program, 2024: The need for a Life Stages approach to triple elimination
Source: PLOS Glob Public Health. 2026 Apr 28;6(4):e0005491. doi: 10.1371/journal.pgph.0005491 (PMC13123933; doi:10.1371/journal.pgph.0005491)
Supplement: Attachment — Submitted filename: PGPH-D-25-03332 with reviewer corrections.docx [file pgph.0005491.s002.docx]

PLOS Global Public Health

Evaluation of Sierra Leone’s Elimination of Mother-to-child Transmission of HIV Program, 2024: The Need for a Life Course Approach to Triple Elimination

--Manuscript Draft--

| Manuscript Number: | PGPH-D-25-03332 |
| --- | --- |
| Article Type: | Research Article |
| Full Title: | Evaluation of Sierra Leone’s Elimination of Mother-to-child Transmission of HIV Program, 2024: The Need for a Life Course Approach to Triple Elimination |
| Short Title: | Rapid evaluation of the Sierra Leone eMTCT program |
| Corresponding Author: | Sulaiman Lakoh  University of Sierra Leone College of Medicine and Allied Health Sciences Freetown, SIERRA LEONE |
| Order of Authors: | Mariama Mustapha |
|  | Ibrahim Turay |
|  | Finda P. Pessima |
|  | Francis K Tamba |
|  | Semion Saffa-Turay |
|  | Gerald Younge |
|  | Veronica L Deen |
|  | Eshetu Kebede Tabor |
|  | Tagoola Abner |
|  | Ginika Egesimba |
|  | Ibrahim Franklyn Kamara |
|  | Basil Uguge |
|  | Godswill Agada |
|  | Silas Quaye |
|  | Tony T Ao |
|  | Andrew Abutu |
|  | Matilda N Kamara |
|  | Osman Sankoh |
|  | Ernest Kenu |
|  | Isaac Ahemesah |
|  | Sartie Kenneh |
|  | Austin Demby |
|  | Sulaiman Lakoh |
| Keywords: | eMTCT, PMTCT, HIV, Sierra Leone, HIV exposed infants, HEI, ANC, antiretroviral therapy, facility delivery, life course approach, ART |
| Abstract: | Background: The elimination of Mother-to-Child Transmission (eMTCT) Cascade Analysis is a key intervention to understand the effectiveness and gaps in national HIV prevention programs for pregnant women and their infants. This comprehensive evaluation aimed to assess the step-by-step processes of eMTCT services, from the entry points at antenatal care (ANC) to outcomes for HIV-exposed infants (HEI) and propose actionable recommendations using the life course approach to improve |

|  | maternal and child health outcomes in Sierra Leone.  Methods: We used a retrospective cohort study to understand the entry of HIV-positive pregnant women into the eMTCT program and the outcome of their infants. The assessment was conducted in June 2024 across 118 selected health facilities nationwide. Data was analysed using Microsoft Excel to assess the steps from entry into the eMTCT program to HIV testing outcomes and delivery process for infants, including final infant/child outcomes for HIV-positive pregnant and breastfeeding women.  Results: Of the 80,264 pregnant women attending their first ANC visit between January and December 2022, 74,401(92.7%) received HIV tests. Of the 2,083 newly diagnosed HIV-positive pregnant women, 1,612 (77.4%) were initiated on antiretroviral therapy (ART) at their first ANC visit, highlighting a critical gap where 22.6% missed early treatment initiation. Only 753 (36.1%) HIV-positive women had documented facility-based delivery, indicating significant drop-offs in retention and continuity of care.  Among 761 HEIs, 671 (88.2%) received ART for prophylaxis within 72 hours of birth; however, only 466 (61.2%) underwent their first virological test within 6-8 weeks of birth, and 176 (24.1%) received a second virological test 12 weeks after cessation of breastfeeding.  Conclusion: While Sierra Leone's eMTCT program has made substantial progress in ANC attendance and HIV testing, critical gaps persist in ART initiation, facility delivery of HIV-positive women and retention in care. Adopting a life course approach is critical to address these gaps through targeted interventions, improved data quality, and enhanced follow-up care. |
| --- | --- |
| Additional Information: |  |
| Question | Response |
| Financial Disclosure  Enter a financial disclosure statement that describes the sources of funding for the work included in this submission and the role the funder(s) played. This includes grants and any commercial funding of the work or authors.  This statement will be typeset if the manuscript is accepted for publication.  Please review the [submission guidelines](http://journals.plos.org/globalpublichealth/s/submission-guidelines#loc-financial-disclosure-statement) and the instructions link below for detailed requirements and guidance. | This work was supported by the Global Fund to fight HIV, Tuberculosis and Malaria and through the Joint United Nations Agency against HIV/AIDS. |
| Competing Interests  On behalf of all authors, disclose any competing interests that could be perceived to bias this work.  This statement will be typeset if the manuscript is accepted for publication. | The authors declare no competing interest |

| Please review the instructions link below and PLOS Global Public Health’s [competing interests](https://journals.plos.org/globalpublichealth/s/competing-interests) policy to determine what information must be disclosed at submission. |  |
| --- | --- |
| Data Availability  Before publication, Authors are required to make fully available and without restriction all data underlying their findings. Please see our [PLOS Data](https://journals.plos.org/globalpublichealth/s/data-availability) [Policy](https://journals.plos.org/globalpublichealth/s/data-availability) page for detailed information on this policy.  A Data Availability Statement, detailing where the data can be accessed, is required at first submission. Insert your Data Availability Statement in the box below.  Please see the [data reporting](https://journals.plos.org/globalpublichealth/s/submission-guidelines#loc-data-reporting) section of our submission guidelines for instructions on what you need to include in your Data Availability Statement.  This statement will be typeset if the manuscript is accepted for publication.  PLOS allows rare exemptions to address legal and ethical concerns. If you have legal or ethical restrictions, please use the box below to detail these in full sentences for the Journal team to consider. | The data supporting this article is available from: <https://figshare.com/articles/dataset/Rapid_eMTCT_casecade_analysis_in_Sierra_Leon> e/30473198?file=59139068. Identifier: 10.6084/m9.figshare.30473198 |

<https://www2.cloud.editorialmanager.com/pgph/download.aspx?id=661251%26guid=99ebda83-10ef-42ed-bb85-0b0eec1355c2%26scheme=1>Manuscript	[Click here to access/download;Manuscript;eMTCT_life couse_paper 9-9-25.docx](https://www2.cloud.editorialmanager.com/pgph/download.aspx?id=661251&guid=99ebda83-10ef-42ed-bb85-0b0eec1355c2&scheme=1)

1 *RESEARCH ARTICLE*

# 2 Evaluation of Sierra Leone’s Elimination of Mother-to-child Transmission of HIV Program, 2024:

3 **The Need for a Life Course Approach to Triple Elimination**
4 Mariama Mustapha^1^, Ibrahim Turay^2^, Finda P. Pessima^2^, Francis K. Tamba^2^, Semion Saffa-Turay^3^,
5 Gerald Younge^2^, Veronica L. Deen^2^, Eshetu Kebede Tabor^2,4^, Tagoola Abner^2^, Ginika Egesimba^2^,
6 Ibrahim Franklyn Kamara^4^, Basil Uguge^5^, Godswill Agada^5^, Silas Quaye^6^, Tony T. Ao^6^, Andrew
7 Abutu^7^, Matilda N. Kamara^8^, Osman Sankoh^9,10^, Ernest Kenu^11^, Isaac Ahemesah^3^, Sartie M.
8 Kenneh^8^, Austin Demby^8^, Sulaiman Lakoh^8,12^
9 ^1^Research Unit, KYM Consultancy Limited, Freetown, Sierra Leone: [mamspha1@gmail.com](mailto:mamspha1@gmail.com)
10 ^2^National AIDS Control Program, Ministry of Health, Government of Sierra Leone: IT ([turayibrahim01@gmail.com](mailto:turayibrahim01@gmail.com)),
11 FP ([findap@yahoo.com](mailto:findap@yahoo.com)), GY ([youngegerald@gmail.com](mailto:youngegerald@gmail.com)), FKT [(ftamba68@gmail.com),](mailto:(ftamba68@gmail.com) EKT([kebedee@who.int](mailto:kebedee@who.int)),
12 GE	([gk200275@gmail.com](mailto:gk200275@gmail.com)),	GA	([godswill.agada@jhpiego.org](mailto:godswill.agada@jhpiego.org)),	TA	([avtagoola@yahoo.com](mailto:avtagoola@yahoo.com)),	VLD
13 [(veronicabackup301@gmail.com)](mailto:(veronicabackup301@gmail.com)
14 ^3^The Joint United Nations Agency against HIV/AIDS, UNAIDS Country Office in Sierra Leone, Freetown, Sierra
15 Leone: SST ([saffaturays@unaids.org](mailto:saffaturays@unaids.org)), IA ([ahemesahi@unaids.org](mailto:ahemesahi@unaids.org)).
16 ^4^World Health Organization, WHO Country Office in Sierra Leone, Freetown, Sierra Leone: IFK ( [ikamara@who.int](mailto:ikamara@who.int)).
17 ^5^Global Reach Program, Jhpiego Sierra Leone, Freetown, Sierra Leone: BU ([basil.uguge@jhpiego.org](mailto:basil.uguge@jhpiego.org)),
18 ^6^US CDC, Division of Global HIV and TB, Accra, Ghana: TTA ([tfa8@cdc.gov](mailto:tfa8@cdc.gov)), SQ [(wej7@cdc.gov).](mailto:(wej7@cdc.gov)
19 ^7^US CDC, Division of Global HIV and TB, Freetown, Sierra Leone: AB ([kdy7@cdc.gov](mailto:kdy7@cdc.gov))
20 ^8^Ministry of Health, Government of Sierra Leone, Freetown, Sierra Leone: AD ([austin.demby@gmail.com](mailto:austin.demby@gmail.com)), SL
21 [(lakoh2009@gmail.com),](mailto:(lakoh2009@gmail.com) SMK ([sartiekenneh@gmail.com)](mailto:sartiekenneh@gmail.com), MNK [(kamaramatilda9198@gmail.com)](mailto:(kamaramatilda9198@gmail.com)
22 ^9^Centre for Health Research and Training, University of Management and Technology, Freetown, Sierra Leone
23 ^10^School of Community Health Sciences, Njala University, Bo Campus, Bo, Sierra Leone
24 email: [osman.sankoh@unimtech.edu.sl](mailto:osman.sankoh@unimtech.edu.sl)
25 [(oasankoh@gmail.com)](mailto:oasankoh@gmail.com).
26 ^11^School of Public Health, University of Ghana, Accra, Ghana: [ekenu@ug.edu.gh.](mailto:ekenu@ug.edu.gh)
27 ^12^College of Medicine and Allied Health Sciences, University of Sierra Leone
28 **Correspondence:** [**lakoh2009@gmail.com,**](mailto:lakoh2009@gmail.com) **+23278287492**
29 **Short title:** Evaluation of the Sierra Leone’s eMTCT program

# 30 Disclaimer

31 *The views expressed in this manuscript do not necessarily reflect the views of the U.S. Centers for*
32 *Disease Control and Prevention or the U.S. Government, Global Fund and the Joint United Nations*
33 *Agency against HIV/AIDS.*

34

# 35 Abstract

36 **Background**: The elimination of Mother-to-Child Transmission (eMTCT) Cascade Analysis is a key
37 intervention to understand the effectiveness and gaps in national HIV prevention programs for
38 pregnant women and their infants. This comprehensive evaluation aimed to assess the step-by-
39 step processes of eMTCT services, from the entry points at antenatal care (ANC) to outcomes for
40 HIV-exposed infants (HEI) and propose actionable recommendations using the life course
41 approach to improve maternal and child health outcomes in Sierra Leone.
42 **Methods**: We used a retrospective cohort study to understand the entry of HIV-positive pregnant
43 women into the eMTCT program and the outcome of their infants. The assessment was
44 conducted in June 2024 across 118 selected health facilities nationwide. Data was analysed using
45 Microsoft Excel to assess the steps from entry into the eMTCT program to HIV testing outcomes
46 and delivery process for infants, including final infant/child outcomes for HIV-positive pregnant
47 and breastfeeding women.
48 **Results**: Of the 80,264 pregnant women attending their first ANC visit between January and
49 December 2022, 74,401(92.7%) received HIV tests. Of the 2,083 newly diagnosed HIV-positive
50 pregnant women, 1,612 (77.4%) were initiated on antiretroviral therapy (ART) at their first ANC
51 visit, highlighting a critical gap where 22.6% missed early treatment initiation. Only 753 (36.1%)
52 HIV-positive women had documented facility-based delivery, indicating significant drop-offs in
53 retention and continuity of care. Among 761 HEIs, 671 (88.2%) received ART for prophylaxis
54 within 72 hours of birth; however, only 466 (61.2%) underwent their first virological test within
55 6-8 weeks of birth, and 176 (24.1%) received a second virological test 12 weeks after cessation of
56 breastfeeding.
57 **Conclusion:** While Sierra Leone's eMTCT program has made substantial progress in ANC
58 attendance and HIV testing, critical gaps persist in ART initiation, facility delivery of HIV-positive
59 women and retention in care. Adopting a life course approach is critical to address these gaps
60 through targeted interventions, improved data quality, and enhanced follow-up care.
61 **Keywords**: eMTCT, PMTCT, HIV, Sierra Leone, HIV exposed infants, HEI, ANC, antiretroviral
62 therapy, facility delivery, life course approach, ART

# 63 Introduction

64 HIV remains a major global public health issue, having claimed an estimated 42.3 million lives to
65 date [1]. Transmission is ongoing in many countries [2]. An estimated 39.9 million people were
66 living with HIV globally at the end of 2023, 65% of whom are in the WHO African Region. In 2023,
67 an estimated 630,000 people died from HIV-related causes, and 1.3 million people became newly
68 infected with HIV [1]. In Sierra Leone, 77,000 people were living with HIV and 1,800 died from
69 AIDS-related illness in 2023 [3].
70 Mother-to-child transmission (MTCT) of HIV is a public health challenge, particularly in regions
71 with high HIV prevalence [4]. The transmission of HIV from an HIV-positive mother to her child
72 can occur during pregnancy, labor, delivery, or breastfeeding [5]. Without intervention, the risk
73 of MTCT ranges from 15% to 45%. However, effective prevention measures can reduce this risk
74 to less than 2% in non-breastfeeding mothers or less than 5% in breastfeeding populations [4,
75 5].
76 Globally, about 90% of children living with HIV acquire their infection from their mothers during
77 pregnancy, birth, and breastfeeding [6]. In Sierra Leone, the program for the elimination of
78 mother-to-child transmission (eMTCT) started in 2004 and has been rolled out to achieve
79 national coverage, which currently stands at only 87% as of December 2023 [7,8]. HIV testing
80 among pregnant women happens in the context of antenatal care (ANC) and during labor and
81 delivery. Sierra Leone has achieved universal ANC coverage at 78.2%, and 98% of women
82 received ANC from a skilled provider at least once in 2019 [9, 10]. This high ANC skilled
83 attendance, however, has not resulted in optimal coverage of HIV testing for pregnant women
84 and follow-up care for HIV-positive mothers and their infants [8].
85 Although Sierra Leone has shown an increase in the percentage of HIV-positive women who were
86 put on ART – from 51% in 2009 to 87% in 2023 – the country is still far from meeting the eMTCT
87 target of 95% [8, 11]. The sub-optimal ART coverage can be attributed to the non-universal HIV
88 testing coverage for all pregnant women, as well as the low coverage of comprehensive eMTCT
89 sites. Furthermore, the country is not doing well in early infant diagnosis (EID). In 2023, the
90 country reported only 9% of infant HIV testing within two months of birth [8]. Data on infant HIV
91 testing at 9 and 18 months are not available, which presents a challenge in determining the final
92 MTCT outcomes and accounting for MTCT during breastfeeding.
93 Conducting a rapid eMTCT cascade analysis can provide critical insights into the effectiveness of
94 the existing eMTCT program in Sierra Leone, measure progress, identify gaps in service delivery
95 and data management, and inform targeted interventions to improve eMTCT service delivery
96 and health care quality and outcomes for mothers and infants.
97 This comprehensive evaluation aimed to assess the step-by-step processes of eMTCT services,
98 from the entry points at ANC to the final outcomes for HIV-exposed infants (HEI) and generate
99 actionable recommendations for improving maternal and child health outcomes. Furthermore,
100 the cascade analysis informed the establishment of a functional ANC sentinel surveillance system
101 in the country.

102

# 103 Materials and Methods

104 **Study design**
105 This retrospective cohort study was implemented to assess the entry of an HIV-positive pregnant
106 woman into the eMTCT program and the outcome of the infants at the point of exit. The eMTCT
107 cascade analysis and ANC site assessment was conducted in June 2024 across selected health
108 facilities nationwide.

# 109 Study setting

110 Sierra Leone is a West African country, bordered by Guinea to the north and east, Liberia to the
111 southeast, and the Atlantic Ocean to the west and southwest. The country has five administrative
112 regions and 16 health districts [10, 12]. Sierra Leone has an estimated population of 8. 7 million
113 with an annual growth rate of 2.13% [12]. It has an estimated infant mortality rate of 70.5 per
114 1,000 live births and an under-five mortality rate of 98.1 per 1,000 live births [12, 13]. The
115 healthcare system in Sierra Leone is under-resourced. It faces numerous challenges, including
116 limited access to healthcare services, a high burden of infectious diseases such as malaria, and
117 infant deaths [14,15]. With funding from the Global Fund, The President's Emergency Plan for
118 Aids Relief (PEPFAR), and other Health Development Partners, eMTCT activities have been scaled
119 up in the past few years in Sierra Leone. There are currently about 960 sites in Sierra Leone that
120 provide eMTCT services but little is known about the uptake of services, acceptance of HIV
121 testing, and the status of syphilis and hepatitis B testing.

# 122 Study population

123 The study included all pregnant women of reproductive age (15-49) who accessed ANC services
124 from January to December 2022. HIV-exposed infants (HEI) of pregnant women in this cohort
125 were included and followed up to determine their final HIV status.

# 126 Sampling procedure

127 We used the UNAIDS/WHO methodology to select a convenient sample of 118 health facilities
128 that reported data on eMTCT variables from January to December 2022 for the cascade analysis
129 and site assessment [16]. The selection of these facilities reflects different geographical locations
130 (urban vs. rural) and HIV burden. The health facilities were distributed across the 16 Sierra Leone
131 districts, including the existing ANC sentinel survey sites. They represent 43% of ANC testing
132 volume and 77% of HIV positivity. Increasing the ANC testing volume did not yield better coverage
133 regarding positivity.
134 We used a cut-off of health facilities that reported 6 HIV-positive pregnant and breastfeeding
135 women in one year. This arbitrary figure of 6 was decided based on the country's routine program
136 data, which indicates a 5-6% HIV positivity rate. Additionally, the cascade analysis tracked
137 variables such as HIV-positive pregnant and breastfeeding women, including exposed infants, to
138 assess service quality and outcomes. Having adequate quantity in each assessed health facility
139 was critical to the assessment/eMTCT cascade analysis.

# 140 Data collection

141 Data was collected using a locally adapted collection tool (Supplementary table 1). Data was
142 abstracted from routine facility registers using three pre-tested data collection tools on June
143 20, 2024 in accordance with national data protection guidelines and ethical standards for
144 secondary data analysis. The authors do not have access to information that could
145 identify individual participants during and after data collection. A hybrid approach was
146 utilized for data collection. Quantitative data elements that were electronically recorded were
147 collected using the Open Data Kit (ODK) software. Using the appropriate registers, we recorded
148 all pregnant women who had their first ANC visit (ANC 1) and were tested for HIV during the
149 specified period. We then created a line list of all clients who tested HIV positive during their first
150 ANC visit to track their progress through delivery and postnatal care. This involved documenting
151 the services the women and their children received, including ART, post-exposure prophylaxis,
152 EID services, and rapid diagnostic test (RDT). Trained teams assessed health facilities' readiness
153 and availability, including collecting and analyzing data using standard tools. The teams
154 collaborated with HIV counsellors, ANC, labour, and post-natal staff at the respective health
155 facilities to complete the assessment. They verified and documented all existing data collection
156 tools and source documents listed in the data abstraction tools. Data entered from the field was
157 synced in real time except during work in areas with poor internet coverage. The central
158 dashboard and data repository systems had access levels to guarantee data security. Paper
159 abstracted data was cross-checked at the end of each day for accuracy and completeness.

# 160 Data analysis

161 The data collected was downloaded to a Microsoft Excel workbook and cleaned by employing
162 processes to query errors, including outliers. The data was then coded, and a pre-developed Excel
163 eMTCT cascade analysis tool was used to analyze data. Data entered into the Excel eMTCT
164 cascade analysis tool was analyzed in steps from entry into the eMTCT program to HIV testing
165 outcomes and delivery of infants, including final infant/child outcomes for HIV-positive pregnant
166 and breastfeeding women.

# 167 Ethical consideration

168 The Sierra Leone Ethics and Scientific Review Committee of the Ministry of Health, Government
169 of Sierra Leone, approved the application for non-research determination prior to the start of the
170 study. The approval number is 014/06/2024. Routine data (including HIV test results) were
171 abstracted under a waiver of informed consent.

172

# 173 Results

174 **ANC site assessment of health facilities with eMTCT program**
175 A total of 118 ANC sites in health facilities was assessed. Of these, 37.3% (44/118) were in the
176 Western Area. On monthly average ANC, 59.3% (70/118) of the facilities had less than 50 ANC
177 attendees. All the 118 facilities perform triple testing (HIV/HBV/Syphilis) using the opt-in approach.
178 Onsite eMTCT HIV testing is nearly evenly split between urban 52.5% (62/118) and rural areas
179 47.5% (56/118), with a slight urban majority. Syphilis testing is routinely done all year, with a slight
180 urban majority of 52.5% (62/118) (Table 1).

# 181 Table 1: Characteristics of health facilities with eMTCT Program, June 2024, Sierra Leone.

| **eMTCT Program Characteristics** | **Residence** | | |
| --- | --- | --- | --- |
|  | **Urban (N = 62) n (%)** | **Rural (N = 56) n (%)** | **Total (N = 118) n (%)** |
| **Region** |  |  |  |
| East | 7 (36.8) | 12 (63.2) | 19 (16.1) |
| North | 5 (45.4) | 6 (54.5) | 11 (9.3) |
| Northwest | 4 (16.7) | 20 (83.3) | 24 (20.3) |
| South | 13 (65.0) | 7 (35.0) | 20 (16.9) |
| West | 33 (75.0) | 11 (25.0) | 44 (37.3) |
| **ANC registrants** |  |  |  |
| < 50 registrants | 30 (42.9) | 40 (57.1) | 70 (59.3) |
| ≥ 50 registrants | 32 (66.7) | 16 (33.3) | 48 (40.7) |
| **PMTCT Testing** |  |  |  |
| Onsite | 62 (52.5) | 56 (47.5) | 118 (100.0) |
| **PMTCT Testing approach** |  |  |  |
| Opt-in | 62 (52.5) | 56 (47.5) | 118 (100.0) |
| **Syphilis testing** |  |  |  |
| Syphilis testing done routinely all | 62 (52.5) | 56 (47.5) | 118 (100.0) |
| year |  |  |  |
| **Nature of syphilis Testing** |  |  |  |
| Onsite | 62 (53.0) | 55 (47.0) | 117 (99.2) |
| Offsite | 0 (0.0) | 1(1.8) | 1 (0.8) |
| **HBV testing part of routine ANC** |  |  |  |
| HBV testing done routinely all year | 24 (72.7) | 9 (27.3) | 33 (28.0) |

| HBV testing done routinely only | 3 (100.0) | 0 (0.0) | 3 (2.5) |
| --- | --- | --- | --- |
| during surveillance |  |  |  |
| HBV testing not done routinely | 35 (42.7) | 47 (57.3) | 82 (69.5) |
| **Nature of HBV Testing** |  |  |  |
| Onsite | 34 (81.0) | 8 (19.0) | 42 (35.6) |
| Offsite | 28 (36.8) | 48 (63.2) | 76 (64.4) |

182 *ANC-Antenatal Clinic, eMTCT-elimination of mother-to-child transmission, HBV-Hepatitis B Virus, PMTCT-Prevention*
183 *of Mother-to-Child Transmission*

# 184 Logistics availability and payment for testing services

185 Out of the 118 health facilities assessed, 35.6% (42/118) facilities had the capability to perform
186 triple testing onsite. Most [81.0% (34/42)] of the facilities were located in the urban setting.
187 Regarding payment for eMTCT services, 11.3% (7/62) of the facilities in the urban setting charged
188 for eMTCT services. However, none of the facilities in the rural setting received payment from
189 clients for eMTCT services. HIV test kit stockouts are more frequent in rural areas, 54.8% (40/73)
190 compared to urban settings. In 2023, 59.7% (43/72) of the health facilities with stockout of HIV
191 test kits experienced at least two instances of stockout within the year (Table 2).

192

# 193 Table 2: Logistics for testing services in Health facilities, June 2024, Sierra Leone.

| **Characteristics** | **Residence** | | |
| --- | --- | --- | --- |
|  | **Urban (N = 62)**  **n (%)** | **Rural (N = 56)**  **n (%)** | **Total (N = 118)**  **n (%)** |
| **Payment for eMTCT services** |  |  |  |
| Yes | 7 (11.3) | 0 (0.0) | 7 (5.9) |
| No | 55 (88.7) | 56 (100.0) | 111 (94.1) |
| **HIV Test Kits stockout** |  |  |  |
| Yes | 33 (53.2) | 40 (71.4) | 73 (61.9) |
| No | 29 (46.8) | 16 (28.6) | 45 (38.1) |
| **Instances of stockout** |  |  |  |
| Once | 14 (42.4) | 15 (37.5) | 29 (39.7) |
| Twice | 19 (57.6) | 25 (62.5) | 44 (60.3) |
| **Triple testing** |  |  |  |

| Yes | 34 (54.8) | 8 (14.3) | 42 (35.6) |
| --- | --- | --- | --- |
| No | 28 (45.2) | 48 (85.7) | 76 (64.4) |

194 *eMTCT-elimination of mother-to-child transmission*

# 195 Maternal eMTCT cascade analysis

196 Between January and December 2022, we assessed 80,264 pregnant women who attended the
197 first ANC visit. Of these, 80,264 ANC attendees, 92.7% (74,401/80,264) received HIV testing
198 during their first ANC visit. Of all the newly tested HIV-positive women, 77.4% (1,612/2,083) were
199 initiated on ART at their first ANC visit, and of those that tested positive, 36.1% (753/2,083) had
200 documented facility-based delivery (Figure 1).

# 201 Gaps in the maternal eMTCT cascade

202 The number clients not tested for HIV on their first ANC visit was 5,863 (7.3%). The East region
203 had the highest proportion, 49.5% (2,904/5,863), of ANC attendance not tested for HIV on their
204 first visit, while the Northern region had the lowest proportion, 4.3% (253/2,896).
205 Also, out of the 2,083 HIV positive women, 22.6% (471) were not initiated on ART with Eastern
206 region contributing the largest proportion (31.8%, 150/471). Out of 2, 083 HIV-positive pregnant
207 women, 63.9% (1330/2083) did not deliver at the health facility. The West region had the highest
208 proportion 56.6% (753/1330) of HIV+ women who did not deliver in the health facility (Table 3).

# 209 Table 3: Gaps in maternal eMTCT cascade of health facilities, June 2024, Sierra Leone

| **Region** | **ANC Registrants not tested for HIV**  **(N = 5,863)**  **n (%)** | **HIV+ clients not on ART**  **(N = 471)**  **n (%)** | **HIV+ clients who deliver outside a health facility (N = 1,330)**  **n (%)** |
| --- | --- | --- | --- |
| East | 2,904 (49.5) | 150 (31.8) | 176 (13.2) |
| North | 253 (4.3) | 22 (4.7) | 56 (4.2) |
| Northwest | 1,014 (17.3) | 120 (25.5) | 183 (13.8) |
| West | 1,692 (28.9) | 139 (29.5) | 753 (56.6) |
| South | 0(0.0) | 40 (8.5) | 162 (12.2) |

210 *ANC-antenatal clinic, ART-antiretroviral therapy, HIV-Human immunodeficiency virus*

211

212

# 213 Infant eMTCT cascade analysis

214 Of all the women who tested positive, 36.1% (753/2,083) delivered 761 exposed infants in the
215 health facilities. A high proportion of 88.2% (671/761) of HEIs received NVP prophylaxis within
216 72 hours after birth and 61.2% (466/761) had their first virological test within 6-8 weeks of birth.
217 Only 6.4% (30/466) of the exposed infants tested positive for HIV after the first virological test,
218 and 24.1% (176/731) of HEIs had a second virological test done 12 weeks after cessation of
219 breastfeeding. Of the 176 exposed infants with the second test, 16.5% (29/176) tested positive
220 (Figure 2).

# 221 Gaps in the infant eMTCT cascade

222 Out of 761 exposed infants, 11.8% (90/761) were not provided ARV prophylaxis within 72 hours
223 of birth, 38.8% (295/761) did not receive their first virological test within 6-8 weeks of birth, and
224 75.9% (555/731) were without second virology test. Out of the 106 HIV-positive infants, 6.6%
225 (7/106) were not linked to care (Table 4).

# 226 Table 4: Gaps in infant eMTCT cascade of health facilities, June 2024, Sierra Leone

| **HIV-Exposed Infants not Initiated on ARV (N = 90)**  **n (%)** | | **HIV-Exposed Infants without 1st Virological Test**  **(N = 295) n (%)** | **HIV+ not linked to Care**  **(N = 7)**  **n (%)** | **No 2^nd^ virology test performed (N = 555)**  **n (%)** | **Did Not Receive Test Results (N = 66)**  **n (%)** |
| --- | --- | --- | --- | --- | --- |
| East | 20 (22.2) | 140 (47.5) | 0 | 103 (18.6) | 0 |
| North | 0 | 5 (1.7) | 1 (14.3) | 21 (3.8) | 1 (1.5) |
| Northwe |  |  |  |  |  |
| st | 27 (30.0) | 21 (7.1) | 0 | 80 (14.4) | 7 (10.6) |
| West | 39 (43.3) | 91 (30.8) | 6 (85.7) | 300 (54.5) | 57 (86.4) |
| South | 4 (4.4) | 38 (12.9) | 0 | 51 (9.2) | 1 (1.5) |

227 *ARV-antiretroviral*

228

# 229 Discussion

230 In this first, large scale, eMTCT cascade analysis in Sierra Leone, we highlighted four key findings
231 on the maternal and infant HIV care cascades. First, only 35.6% of the facilities, mostly in urban
232 areas, could perform triple testing. Second, 92.7% of pregnant women received an HIV test at
233 their first antenatal visit and 77.4% of those with a positive result were receiving ART, but only
234 36.1% had documented deliveries in health facilities. Third, 61.2% of the positive newborns
235 underwent the first virological test within 6-8 weeks after birth, 6.4% of which were positive, and
236 24.1% of the newborns underwent the second test 12 weeks after stopping breastfeeding, 16.5%
237 of which were positive. Finally, NVP prophylaxis was missed in 11.8% of HEIs.
238 With the scale-up of eMTCT services across the country in the past several years, an MTCT rate
239 of 16.5% is disappointing as Sierra Leone is far from achieving the global target of 5% in
240 breastfeeding populations and 2% in non-breastfeeding populations [5]. There is need for a
241 concerted and proactive approach among all stakeholders for strategic investment to identify and
242 place all HIV positive women of reproductive age and pregnant women on sustained ART and
243 improve on infant HIV services in line with the current national and international guidelines.
244 While this finding is similar to the UNAIDS reported MTCT rate of 19% for Sierra Leone, it is in
245 contrast with MTCT rate in Kenya (8.9%) and that of Botswana (2.2%), a country that is on the
246 path for elimination of vertical transmission of HIV [17,18].
247 To eliminate mother-to-child transmission of HIV, women and their infants must be enrolled and
248 retained in the eMTCT continuum of care from ANC through delivery and 18 to 24 months post-
249 partum. However, many women in our study failed to enroll in care despite several opportunities
250 and many others dropped out or are lost to follow-up in the eMTCT care continuum. The reason
251 why pregnant women drop out of treatment is unclear but may be due to lack of follow-up
252 services for HIV pregnant women or poor health literacy among the HIV positive women.
253 In March 2024, WHO published the triple elimination framework to encourage countries to
254 address the gaps in vertical transmission of HIV, hepatitis B, and syphilis, including testing [4]. Our
255 study shows that most health facilities in Sierra Leone do not have the capacity to implement the
256 triple elimination, underscoring the need for training, mentoring, and supportive supervision.
257 The Ministry of Health and its partners have begun implementing triple elimination using a life
258 course approach to ensure that the health needs of our children are addressed throughout their
259 life course. We will strengthen the elimination of these three diseases in line with Sierra Leone’s
260 framework of holistic care to HEIs using the country’s life course approach to prevent future
261 vulnerabilities [19].
262 Despite the progress in HIV testing of pregnant women in Sierra Leone, the 93% of the ANC
263 attendees tested for HIV between January and December 2022 was lower than the 96.3%
264 reported in Ghana and 98.5% reported in Tanzania [20, 21]. ANC registration is the first point of
265 contact that allows attendees to be recruited into the cascade based on their test results. This
266 implies that the 7% untested ANC attendees in this study were not recruited into the cascade,
267 denying them the benefits of knowing their HIV status and all the measures to improve their
268 health and eliminate HIV transmission to their babies. The reasons for this gap are unclear but
269 could be investigated in future research. However, to achieve a 100% testing rate for ANC
270 attendees, it is essential to intensify education and individual-level counselling and strengthen
271 HIV surveillance among pregnant women. One of the established approaches is the use of
272 mentor mothers in following up pregnant women and HEIs until the final infant outcome [22]. We
273 will explore the use of mentor mothers to assign cohorts of pregnant women for consistent
274 follow-up of the final infant outcome.
275 Less than 80% of pregnant women who tested positive for HIV were initiated on ART at their first
276 ANC visit. Although this is not unique to Sierra Leone, it reflects a worrying trend in the cascade
277 since more than 20% of those who tested positive still left the cascade. Without ART, the viral
278 load in an HIV-positive pregnant woman remains high, significantly increasing the risk of
279 transmitting the virus to the baby in utero, thereby thwarting the efforts made in the country’s
280 attempt to eMTCT [4]. These women are also at higher risk for pregnancy-related complications
281 such as preterm labor, low birth weight, and stillbirth. Historically, Tanzania reported similar ART
282 uptake (82%) among women who tested positive for HIV in 2017 [21]. In Kenya, the rate of ART
283 uptake among eligible pregnant women reported in 2012 is much lower than we reported (only
284 40% in Naivasha and 27% in Gilgil) [23]. However, it is difficult to make a direct comparison of
285 findings from these studies with our results due to the time laps, underscoring the importance of
286 strengthening counselling and linkage to treatment, care, and support services in Sierra Leone.
287 While Sierra Leone had over 83% of deliveries in health facilities, the proportion of documented
288 facility-based delivery in our study is unacceptably low at 36%. Clients who deliver outside of
289 health facilities usually miss out on maternal and infant interventions, including zidovudine (AZT)
290 and NVP prophylaxis for HEI [24], thereby increasing the risk of MTCT of HIV [25]. The low rate of
291 facility deliveries may be due to stigma, which leads women to be delivered in facilities where
292 they were not tested for HIV. We recommend using a biometric system and unique identifiers to
293 track pregnancies, no matter where they deliver, so their children can benefit from NVP and AZT
294 prophylaxis. Furthermore, Sierra Leone is implementing community-led monitoring across several
295 districts to gather quantitative and qualitative data about HIV services [26]. This program will be
296 expanded and tailored to all the districts to reduce stigma and other barriers to the low hospital
297 delivery rate among pregnant women with HIV in Sierra Leone.
298 Along the infant care cascade, about 88% of HEIs received NVP prophylaxis within 72 hours after
299 birth, implying that more than 10% of infants are without ARV prophylaxis. NVP is given to the
300 infant immediately after birth to protect against the virus during this high-risk period. Failure to
301 give the drug increases the risk of transmission in the infant [24]. The number of HEI that missed
302 NVP prophylaxis in our study is lower than reported in Mulago National Referral Hospital in
303 Uganda in 2024 (11.8% vs. 21.9%) [27].
304 Despite the high rate of initial prophylaxis, 61.2% of the HEIs underwent their first virological test
305 within 6-8 weeks of birth, revealing gaps in early diagnostic follow-up. Moreover, 6% of HEIs
306 tested positive for HIV. This is more than 3.6% reported in Nigeria, indicating potential
307 transmission during pregnancy or delivery [28]. Furthermore, less than 25% of the infants
308 received a second virological test 12 weeks after the cessation of breastfeeding, with 16.5%
309 showing HIV transmission at this stage. This highlights the need for continued vigilance and
310 repeated testing to ensure early detection and intervention [29]. Mobile phone calls reportedly
311 improve maternal and infant adherence to eMTCT services in western Kenya, and this service will
312 be strengthened in Sierra Leone to address gaps in eMTCT [30]. To ensure these measures are as
313 effective as they should be, the Ministry of Health and its partners will address frequent
314 commodity stock-outs and strengthen laboratory systems. Despite improvements in eMTCT
315 programs, gaps in follow-up testing and treatment adherence continue to impact HIV

316

317

318

319

320

321

322

323

324

325

326

327

328

329

330

331

332

333

334

335

336

337

338

339

340

341

342

transmission rates among infants [31]. Studies in Zimbabwe have also shown that while initial NVP prophylaxis uptake is high, follow-up testing rates drop significantly, mirroring the trends observed in this study [32].

Our study has several strengths. First, this is the first large-scale nationwide study conducted to assess the eMTCT cascade in Siera Leone. Therefore, the findings can be generalized. Second, the data collection tools were validated, thereby refining complex questions and ensuring uniform

data collection. Healthcare professionals with expertise in reading patients' notes and understanding the different terminologies collected the data. This ensures efficient data collection . Finally, we followed the strengthening reporting of observational study (STROBE) guidelines to report our findings.

Although this assessment provides a detailed analysis of the country’s performance on key eMTCT indicators, it has some limitations. This dataset is based on the records from routine services that may sometimes be incomplete. Also, since this data does not include sociodemographic information on clients, it is difficult to determine the association between attrition from the cascade and patient characteristics.

# Conclusion

While Sierra Leone's eMTCT program has made substantial progress, particularly in the early stages of the cascade, such as ANC attendance and HIV testing, there are critical gaps in ART initiation, delivery of HIV-positive women in health facilities, and retention in care of HEIs. A life course approach is essential to addressing these gaps through targeted interventions, improved

data quality, and comprehensive follow-up towards achieving the eMTCT goals and improving health outcomes for mothers and their children in Sierra Leone.

# Supplementary information

Supplementary table 1: Rapid EMTCT Cascade Analysis Tool and Data Sources

# Acknowledgements

We appreciate the support provided by the adm_1_i_4_nistrations of the health facilities where the studies were conducted. We are grateful to the data collectors.

| 343 | **Abbreviations** |  |
| --- | --- | --- |
| 344 | ANC | Antenatal Care |
| 345 | ART | Antiretroviral Treatment |
| 346 | EID | Early Infant Diagnosis |
| 347 | eMTCT | Elimination of Mother-to-Child Transmission |
| 348 | HEI | HIV-exposed infants |
| 349 | HIV | Human Immunodeficiency Viruses |
| 350 | NVP | Nevirapine |
| 351 | PMTCT | Prevention of Mother-to-Child Transmission |
| 352 | PCR | Polymerase Chain Reaction |
| 353 | UNAIDS | The Joint United Nations Agency against HIV/AIDS |
| 354 | WHO | World Health Organization |
| 355 |  |  |

# 356 Authors’ contribution

357 Conceptualization and study design: SL, EKT, TA, SQ, TTA, VLD, IA, EK, GA, BU, SMK, AD and GY
358 Data curation and validation: IT, EK, MM, OS, and FKT
359 Methodology and formal analysis: FPP, IT, MM, SST, IA, SL, FKT, EK, and TA
360 Funding acquisition, resources, supervision: IA, SST, SL, AD, SMK
361 Writing – original draft preparation: SL, EK, MM, MNK, AB, GE, and OS
362 Writing – review & editing: SL, EK, MM, MNK, IFK, AB, GE, and OS

363

364

365

366

All authors read and approved the final manuscript.

# 367 Funding

368 This work is part of the Global Fund support to Sierra Leone through funds outsourced to the
369 Joint United Nations Agency against HIV/AIDS (UNAIDS) Country Office.

# 370 Availability of data and materials

371 The	data	supporting	this	article	is	available	from:
372 [https://figshare.com/articles/dataset/Rapid_eMTCT_casecade_analysis_in_Sierra_Leone/30473](https://figshare.com/articles/dataset/Rapid_eMTCT_casecade_analysis_in_Sierra_Leone/30473198?file=59139068)
373 [198?file=59139068](https://figshare.com/articles/dataset/Rapid_eMTCT_casecade_analysis_in_Sierra_Leone/30473198?file=59139068). Identifier: 10.6084/m9.figshare.30473198

374

# 375 Consent for publication

376 Not applicable

# 377 Competing interests

378 AD is the Minister of Health for Sierra Leone. All the authors declare that they have no competing
379 interests.

380

381

382

# 383 References

384 1. WHO. (2023). *Global HIV Programme*. Available at: [https://www.who.int/teams/global-](https://www.who.int/teams/global-hiv-hepatitis-and-stis-programmes/hiv/prevention/mother-to-child-transmission-of-hiv)
385 [hiv-hepatitis-and-stis-programmes/hiv/prevention/mother-to-child-transmission-of-hiv](https://www.who.int/teams/global-hiv-hepatitis-and-stis-programmes/hiv/prevention/mother-to-child-transmission-of-hiv).
386 Accessed September 1, 2024
387 2. Global	AIDS	Update	(2024)-	AIDS	AT	A	CROSSROADS.	Available	at:
388 [https://www.unaids.org/sites/default/files/media_asset/2024-unaids-global-aids-](https://www.unaids.org/sites/default/files/media_asset/2024-unaids-global-aids-update-summary_en.pdf)
389 [update-summary_en.pdf](https://www.unaids.org/sites/default/files/media_asset/2024-unaids-global-aids-update-summary_en.pdf). Accessed September 26, 2024.
390 3. WHO (2024) HIV Facts Sheet-Available at: [www.who.int](http://www.who.int/). Accessed September 1, 2024.
391 4. WHO-Introducing a framework for implementing triple elimination of mother-to-child
392 transmission	of	HIV,	syphilis	and	hepatitis	B	virus-Available	at:
393 [https://www.who.int/initiatives/triple-elimination-initiative-of-mother-to-child-](https://www.who.int/initiatives/triple-elimination-initiative-of-mother-to-child-transmission-of-hiv-syphilis-and-hepatitis-b.%20Accessed%20September%2015)
394 [transmission-of-hiv-syphilis-and-hepatitis-b. Accessed September 15](https://www.who.int/initiatives/triple-elimination-initiative-of-mother-to-child-transmission-of-hiv-syphilis-and-hepatitis-b.%20Accessed%20September%2015), 2024
395 5. Hamilton, E., Bossiky, B., Ditekemena, J., Esiru, G., Ob, Mm., Srhr, D., Fwamba, F., Goga, A.
396 E., Pat Kieffer, M., Tsague, L. D., van de Ven, R., Wafula, R., & Guay, L. (2017). *Using the*
397 *PMTCT Cascade to Accelerate Achievement of the Global Plan Goals*. [www.jaids.com](http://www.jaids.com/)
398 6. UNODC. (2018). *Prevention of mother-to-child transmission of HIV, hepatitis B and C and*
399 *syphilis.	Available	at:*
400 [https://www.google.com/search?q=UNODC.+(2018).+Prevention+of+mother-to-](https://www.google.com/search?q=UNODC.%2B(2018).%2BPrevention%2Bof%2Bmother-to-child%2Btransmission%2Bof%2BHIV%2C%2Bhepatitis%2BB%2Band%2BC%2Band%2Bsyphilis.&oq=UNODC.%2B(2018).%2BPrevention%2Bof%2Bmother-to-child%2Btransmission%2Bof%2BHIV%2C%2Bhepatitis%2BB%2Band%2BC%2Band%2Bsyphilis.&gs_lcrp=EgZjaHJvbWUyBggAEEUYOdIBCDE2NjBqMGo3qAIAsAIA&sourceid=chrome&ie=UTF-8)
401 [child+transmission+of+HIV%2C+hepatitis+B+and+C+and+syphilis.&oq=UNODC.+(2018).+](https://www.google.com/search?q=UNODC.%2B(2018).%2BPrevention%2Bof%2Bmother-to-child%2Btransmission%2Bof%2BHIV%2C%2Bhepatitis%2BB%2Band%2BC%2Band%2Bsyphilis.&oq=UNODC.%2B(2018).%2BPrevention%2Bof%2Bmother-to-child%2Btransmission%2Bof%2BHIV%2C%2Bhepatitis%2BB%2Band%2BC%2Band%2Bsyphilis.&gs_lcrp=EgZjaHJvbWUyBggAEEUYOdIBCDE2NjBqMGo3qAIAsAIA&sourceid=chrome&ie=UTF-8)
402 [Prevention+of+mother-to-](https://www.google.com/search?q=UNODC.%2B(2018).%2BPrevention%2Bof%2Bmother-to-child%2Btransmission%2Bof%2BHIV%2C%2Bhepatitis%2BB%2Band%2BC%2Band%2Bsyphilis.&oq=UNODC.%2B(2018).%2BPrevention%2Bof%2Bmother-to-child%2Btransmission%2Bof%2BHIV%2C%2Bhepatitis%2BB%2Band%2BC%2Band%2Bsyphilis.&gs_lcrp=EgZjaHJvbWUyBggAEEUYOdIBCDE2NjBqMGo3qAIAsAIA&sourceid=chrome&ie=UTF-8)
403 [child+transmission+of+HIV%2C+hepatitis+B+and+C+and+syphilis.&gs_lcrp=EgZjaHJvbW](https://www.google.com/search?q=UNODC.%2B(2018).%2BPrevention%2Bof%2Bmother-to-child%2Btransmission%2Bof%2BHIV%2C%2Bhepatitis%2BB%2Band%2BC%2Band%2Bsyphilis.&oq=UNODC.%2B(2018).%2BPrevention%2Bof%2Bmother-to-child%2Btransmission%2Bof%2BHIV%2C%2Bhepatitis%2BB%2Band%2BC%2Band%2Bsyphilis.&gs_lcrp=EgZjaHJvbWUyBggAEEUYOdIBCDE2NjBqMGo3qAIAsAIA&sourceid=chrome&ie=UTF-8)
404 [UyBggAEEUYOdIBCDE2NjBqMGo3qAIAsAIA&sourceid=chrome&ie=UTF-8](https://www.google.com/search?q=UNODC.%2B(2018).%2BPrevention%2Bof%2Bmother-to-child%2Btransmission%2Bof%2BHIV%2C%2Bhepatitis%2BB%2Band%2BC%2Band%2Bsyphilis.&oq=UNODC.%2B(2018).%2BPrevention%2Bof%2Bmother-to-child%2Btransmission%2Bof%2BHIV%2C%2Bhepatitis%2BB%2Band%2BC%2Band%2Bsyphilis.&gs_lcrp=EgZjaHJvbWUyBggAEEUYOdIBCDE2NjBqMGo3qAIAsAIA&sourceid=chrome&ie=UTF-8).	Accessed
405 September 1, 2024.
406 7. WHO. (2018). *Country progress report-Sierra Leone Global AIDS Monitoring 2018*.
407 Available at: [https://www.ecoi.net/en/file/local/1441181/1226_1534928713_sle-2018-](https://www.ecoi.net/en/file/local/1441181/1226_1534928713_sle-2018-countryreport.pdf)
408 [countryreport.pdf](https://www.ecoi.net/en/file/local/1441181/1226_1534928713_sle-2018-countryreport.pdf). Accessed September 1, 2024.
409 8. UNAIDS	Facts	sheet	(2023)-Available	at:
410 <https://www.unaids.org/en/regionscountries/countries/sierraleone>.	Accessed
411 September 12, 2024.
412 9. Belay, W., Belay, A., Mengesha, T. *et al.* Demographic and economic inequality of antenatal
413 care coverage in 4 African countries with a high maternal mortality rate. *Arch Public*
414 *Health* **82**, 61 (2024). <https://doi.org/10.1186/s13690-024-01288-3>
415 10. SLDHS. (2019). *Sierra Leone Demographic and Health Survey 2019. Freetown, Sierra*
416 *Leone,	and	Rockville,	Maryland,	USA:	Stats	SL	and	ICF*.	Accessed	on:
417 <https://www.statistics.sl/images/StatisticsSL/Documents/DHS2018/sldhs2019kir.pdf>.
418 11. NAS	Report-Available	at:	<https://www.nas.gov.sl/interventions/pmtct>.	Accessed
419 September 1, 2024.
420 12. Sierra	Leone	Population	(2024)-Available	at:
421 <https://worldpopulationreview.com/countries/sierra-leone>. Accessed on September 1,
422 2024.
423 13. Statistics Sierra Leone. *2015 Population and Housing Census. Summary of Final*
424 *Results.* Statistics	Sierra	Leone;	Freetown,	Sierra	Leone:	Available	at:
425 [https://www.statistics.sl/images/StatisticsSL/Documents/final-results_-](https://www.statistics.sl/images/StatisticsSL/Documents/final-results_-2015_population_and_housing_census.pdf)
426 [2015_population_and_housing_census.pdf](https://www.statistics.sl/images/StatisticsSL/Documents/final-results_-2015_population_and_housing_census.pdf).
427 14. Sierra Leone - Mortality Rate, Infant, Female (per 1,000 Live Births). Available at:
428 [https://tradingeconomics.com/sierra-leone/mortality-rate-infant-female-per-1000-live-](https://tradingeconomics.com/sierra-leone/mortality-rate-infant-female-per-1000-live-births-wb-data.html)
429 [births-wb-data.html](https://tradingeconomics.com/sierra-leone/mortality-rate-infant-female-per-1000-live-births-wb-data.html). Accessed September 4, 2024.
430 15. WHO	Sierra	Leone	Annual	Report-2024.	Available	at:
431 [https://www.afro.who.int/sites/default/files/2024-](https://www.afro.who.int/sites/default/files/2024-04/WHO%20Sierra%20Leone%20Annual%20Report%202023%20Final.pdf)
432 [04/WHO%20Sierra%20Leone%20Annual%20Report%202023%20Final.pdf](https://www.afro.who.int/sites/default/files/2024-04/WHO%20Sierra%20Leone%20Annual%20Report%202023%20Final.pdf).	Accessed
433 September 4, 2024.
434 16. UNAIDS/WHO-Guidelines for Conducting HIV Sentinel Serosurveys among Pregnant
435 Women and Other Groups. Available at: [https://data.unaids.org/publications/irc-](https://data.unaids.org/publications/irc-pub06/jc954-anc-serosurveys_guidelines_en.pdf)
436 [pub06/jc954-anc-serosurveys_guidelines_en.pdf](https://data.unaids.org/publications/irc-pub06/jc954-anc-serosurveys_guidelines_en.pdf). Accessed September 3, 2024.
437 17. UNAIDS. HIV estimates with uncertainty bounds 1990-present. 2023. Available at:
438 [https://www.unaids.org/en/resources/documents/2023/HIV_estimates_with_uncertain](https://www.unaids.org/en/resources/documents/2023/HIV_estimates_with_uncertainty_bounds_1990-present)
439 [ty_bounds_1990-present](https://www.unaids.org/en/resources/documents/2023/HIV_estimates_with_uncertainty_bounds_1990-present). Accessed October 6, 2024.
440 18. UNAIDS. Botswana leads the way for high HIV burden country certification on the path to
441 eliminate	vertical	HIV	transmission	(2022).	Available	at:
442 [https://www.unaids.org/en/resources/presscentre/featurestories/2022/july/20220727_](https://www.unaids.org/en/resources/presscentre/featurestories/2022/july/20220727_botswana-leads-way-eliminate-vertical-HIV-transmission)
443 [botswana-leads-way-eliminate-vertical-HIV-transmission](https://www.unaids.org/en/resources/presscentre/featurestories/2022/july/20220727_botswana-leads-way-eliminate-vertical-HIV-transmission). Accessed October 6, 2024.
444 19. The Sierra Leone framework for the person-centred life stages approach to health service
445 delivery	(2023-2030)-	Available	at:	file:///C:/Users/PC/Downloads/Sierra-Leone-
446 Framework-For-The-Person-Centred-Life-Stages-Approach-to-Service-
447 Delivery%20(2).pdf. Accessed September 13, 2024.
448 20. Abraham, S. A., & Clow, S. E. (2022). Staying, leaving and returning: Trends of prevention
449 of mother-to-child transmission retention among newly diagnosed HIV-positive pregnant
450 and postpartum women. *International Journal of STD and AIDS*, *33*(1), 81–87.
451 <https://doi.org/10.1177/09564624211046995>
452 21. Gamell, A., Luwanda, L. B., Kalinjuma, A. V., Samson, L., Ntamatungiro, A. J., Weisser, M.,
453 Gingo, W., Tanner, M., Hatz, C., Letang, E., & Battegay, M. (2017). Prevention of mother-
454 to-child transmission of HIV Option B+ cascade in rural Tanzania: The One Stop Clinic
455 model. PLoS ONE, 12(7). <https://doi.org/10.1371/journal.pone.0181096>
456 22. Sam-Agudu NA, Cornelius LJ, Okundaye JN, Adeyemi OA, Isah HO, Wiwa OM, Adejuyigbe
457 E, Galadanci H, Afe AJ, Jolaoso I, Bassey E, Charurat ME. The impact of mentor mother
458 programs on PMTCT service uptake and retention-in-care at primary health care facilities
459 in Nigeria: a prospective cohort study (MoMent Nigeria). J Acquir Immune Defic Syndr.
460 2014 Nov 1;67 Suppl 2: S132-8. doi: 10.1097/QAI.0000000000000331. PMID: 25310119.
461 23. Ferguson, L., Lewis, J., Grant, A. D., Watson-Jones, D., Vusha, S., Ong, J. O., & Ross, D. A.
462 (2012).	IMPLEMENTATION	AND	OPERATIONAL	RESEARCH:	EPIDEMIOLOGY	AND
463 PREVENTION Patient Attrition Between Diagnosis With HIV in Pregnancy-Related Services
464 and Long-Term HIV Care and Treatment Services in Kenya: A Retrospective Study.
465 [www.jaids.com](http://www.jaids.com/)
466 24. Podzamczer D, Fumero E. The role of nevirapine in the treatment of HIV-1 disease. Expert

467	Opin Pharmacother. 2001 Dec;2(12):2065-78. doi: 10.1517/14656566.2.12.2065. PMID:

468	11825335.

469 25. Sibanda, E. L., Weller, I. V.D., Hakim, J. G., & Cowan, F. M. (2013). The magnitude of loss to
470 follow-up of HIV-exposed infants along the prevention of mother-to-child HIV
471 transmission continuum of care: A systematic review and meta-analysis. In *AIDS* (Vol. 27,
472 Issue 17, pp. 2787–2797). <https://doi.org/10.1097/QAD.0000000000000027>
473 26. Community	Led	Monitoring	Factsheet	(2020).	Available	at:	chrome-
474 extension://efaidnbmnnnibpcajpcglclefindmkaj/<https://www.state.gov/wp->
475 content/uploads/2020/07/PEPFAR_Community-Led-Monitoring_Fact-Sheet_2020.pdfn.
476 Accessed October 5, 2024.
477 27. Hellen N, Joseph R, Ezekiel M, Fred S, Ronald S, Philippa M. Proportion of HIV exposed
478 infants aged 0-6 months that missed nevirapine prophylaxis in Mulago National Referral
479 Hospital, Uganda: a cross-sectional study. BMC Pediatr. 2024 Jul 4;24(1):430. doi:
480 10.1186/s12887-024-04600-w. PMID: 38965487.
481 28. Rawizza, H. E., Chang, C. A., Chaplin, B., Ahmed, I. A., Meloni, S. T., Oyebode, T., Banigbe,
482 B., Sagay, A. S., Adewole, I. F., Kanki, P. J., & Team, A. P. (n.d.). *Loss to Follow-up within the*
483 *Prevention of Mother-to-Child Transmission Care Cascade in a Large ART Program in*
484 *Nigeria*.
485 29. Ababa, A. (2015). *Report on the 2014 Round Antenatal Care based Sentinel HIV*
486 *Surveillance in Ethiopia*.
487 30. Kebaya LMN, Wamalwa D, Kariuki N, Admani B, Ayieko P, Nduati R. Efficacy of Mobile
488 phone use on adherence to Nevirapine prophylaxis and retention in care among the HIV-
489 exposed infants in prevention of mother to child transmission of HIV: a randomized
490 controlled trial. BMC Pediatr. 2021 Apr 20;21(1):186. doi: 10.1186/s12887-021-02660-w.
491 PMID: 33879118; PMCID: PMC8056659.
492 31. Dinh, T. H., Delaney, K. P., Goga, A., Jackson, D., Lombard, C., Woldesenbet, S., Mogashoa,
493 M., Pillay, Y., & Shaffer, N. (2015). Impact of maternal HIV seroconversion during pregnancy
494 on early mother to child transmission of HIV (MTCT) measured at 4-8 weeks postpartum
495 in South Africa 2011-2012: A national population-based evaluation. *PLoS ONE*, *10*(5).
496 <https://doi.org/10.1371/journal.pone.0125525>
497 32. Kurewa, N. E., Gumbo, F. Z., Mapingure, P. M., Munjoma, M. W., Chirenje, M. Z.,
498 Rusakaniko, S., & Stray-pedersen, B. (2012). Predictors of attrition among children born in
499 a PMTCT programme in Zimbabwe followed up over 5 years. Journal of Tropical Pediatrics,
500 58(5), 360–369. <https://doi.org/10.1093/tropej/fmr112>

501

Figure 1	[Click here to access/download;Figure;Figure 1 eMTCT.docx](https://www2.cloud.editorialmanager.com/pgph/download.aspx?id=659900&guid=a6f46e24-296a-4a47-8d69-e7d78d9d19df&scheme=1)[<https://www2.cloud.editorialmanager.com/pgph/download.aspx?id=659900%26guid=a6f46e24-296a-4a47-8d69-e7d78d9d19df%26scheme=1>](https://www2.cloud.editorialmanager.com/pgph/download.aspx?id=659900&guid=a6f46e24-296a-4a47-8d69-e7d78d9d19df&scheme=1)

Proportion of women

*ANC-antenatal care	ART-antiretroviral therapy, HIV-Human Immunodeficiency Virus*

120

100

100

92.7

80

77.4

60

40

36.1

20

0

Number of pregnant HIV Testing at ANC	HIV+ women newly HIV+ women deliver

women with 1st ANC	1st visit

diagnosed and initiated on ART at ANC1

Cascade stages

at health facility

**Figure 1: Maternal eMTCT cascade of health facilities, June 2024, Sierra Leone**

<https://www2.cloud.editorialmanager.com/pgph/download.aspx?id=659901%26guid=a68ce53f-0c70-4bf6-98b8-000417692a5b%26scheme=1>Figure 2	[Click here to access/download;Figure;Figure 2 eMTCT manuscript.docx](https://www2.cloud.editorialmanager.com/pgph/download.aspx?id=659901&guid=a68ce53f-0c70-4bf6-98b8-000417692a5b&scheme=1)

Proportion of infants

**Figure 2: Infant cascade MTCT cascade of health facilities, June 2024, Sierra Leone**

120.0

101.1

100.0

88.2

80.0

61.2

60.0

40.0

24.1

29.9

20.0

16.5

20.6

6.4

0.0

No. of HIV exposed infants born to the HIV + women

No. of HIV exposed infants initiated on ARV

prophylaxis (NVP) within

No. of HIV	Infants	No. of HIV	Infants	No. of HIV exposed	identified as	exposed	identified as	exposed infants with	HIV +	infants with	HIV + (2nd	infants with

1st virological (reactive 1st 2nd virological virological test HIV Rapid

Children identified as HIV + (Rapid tests)

test (DNA virological test test (DNA	result)	Antibody Test

PCR) done	result)	PCR) done 12

within 6-8 72 hours of weeks of birth

birth

weeks after cessation of breastfeeding or as indicated

done at 18 months or thereafter)
