# Supplementary material for: Evaluation of Sierra Leone’s Elimination of Mother-to-Child Transmission of HIV program, 2024: The need for a Life Stages approach to triple elimination
Source: PLOS Glob Public Health. 2026 Apr 28;6(4):e0005491. doi: 10.1371/journal.pgph.0005491 (PMC13123933; doi:10.1371/journal.pgph.0005491)
Supplement: Attachment — Submitted filename: eMTCT response to reviewers_15.03.26.docx [file pgph.0005491.s003.docx]

PGPH-D-25-03332
Evaluation of Sierra Leone’s Elimination of Mother-to-child Transmission of HIV Program, 2024: The Need for a Life Course Approach to Triple Elimination

**Responses to reviewer 1**

The manuscript is well structured and easy to follow

Thank you.

The manuscript provides a comprehensive evaluation of Sierra Leone’s Elimination of Mother-to-Child Transmission of HIV (eMTCT) Program. The data presented are crucial for understanding the current state of HIV prevention efforts in the country, and the analysis highlights both progress and significant gaps. While the study successfully outlines key findings, there are areas that require further elaboration and refinement to enhance clarity and impact.

Thank you. We appreciate this nice comment on our paper

The introduction effectively sets the context but could be further improved by clearly defining terms such as eMTCT early on. Additionally, the objectives of the study should be explicitly stated as separate bullet points to improve readability. (Paragraphs 63-75)

Thank you. We have added information on the definition of eMTCT in lines 79-82 of the revised manuscript. However, we feel that the objective should not be stated in bullet points. We believe that the objectives of this study are well-defined and easy to understand (lines 101-104).

Methodology: (Paragraphs 103-171)
The methodology section is generally well-structured; however, it lacks details on the ethical considerations taken during data collection, particularly regarding consent for secondary data. Including a brief section on ethical compliance and how participant confidentiality was ensured would strengthen this section.

Thank you for your valuable feedback. We have added more information on the ethical consideration section, including data confidentiality (lines 171-176).

Results Presentation (Paragraphs 174-225):
The results are well-documented; however, the tables could be visually enhanced for better comprehension. Consider using color coding or differing formats to differentiate categories. Furthermore, the gaps identified in the results (e.g., ART initiation, facility-based deliveries) should be discussed in more detail, exploring the underlying causes.

We fill it will be odd to use color coding or differing format to differentiate categories. It is unlikely that the journal will publish tables with differing format or differing color coding. Nonetheless, we have changed the color of figure 1 to a brighter blue color. We added information on ART initiation, exploring factors such as poor psychosocial counselling in lines 290 to 292 of the revised manuscript. We extensively discussed facility deliveries in lines 303 to 313.

Discussion and Context (Paragraphs 230-310):
o While the discussion addresses the implications of the findings, it could benefit from additional comparative analysis with other countries. Incorporating specific examples of successful interventions from similar contexts could provide valuable insights for stakeholders in Sierra Leone. Additionally, expanding on systemic barriers to care identified in the results would enrich this section.

Thank you! we added details on this in the discussion (lines 251 to 262)

5. Conclusion and Recommendations (Paragraphs 332-337):
o The conclusion succinctly summarizes findings but lacks specific, actionable recommendations tailored for various stakeholders. Include a clear call to action, emphasizing the role of different entities, such as government agencies, healthcare providers, and community organizations, in implementing changes based on the findings.

Thank you! We have added details “Our findings indicate the need for radical measures for health facilities to test every pregnant woman for HIV, HBV, and syphilis, and to link every pregnant woman who tests positive with treatment, care, and support services. Second, we recommend that community actors (such as mentor-mothers) follow up on all pregnant women living with HIV until they have a documented delivery in a medical facility. Finally, all HEI patients should receive antiretroviral prophylaxis according to national guidelines and undergo nucleic acid amplification testing within 6 to 8 weeks after birth” in the conclusion.

6. Data Integrity and Analysis(see Paragraphs 161-170):
o The use of descriptive statistics is appropriate, but more detailed statistical analyses (e.g., confidence intervals, significance tests) would lend credibility to the findings. Consider addressing potential biases in data collection and how they might affect the results.

This study assesses various outcomes and has provided a detailed description of the eMTCT cascade analysis in Sierra Leone. The gaps identified in this study are now been addressed by stakeholders in the country. We added the limitations of using secondary data in the limitation section in line 354 to 356.

7. Future Research Directions (End of Discussion) :
o The manuscript would benefit from a section outlining future research needs. This could include recommendations for longitudinal studies to track long-term outcomes for HIV-exposed infants or assessments of specific interventions aimed at improving ART uptake.

We have added these details in lines 342 to 346 of the manuscript

Additional Considerations:
• Ethics and Dual Publication:
o There are no apparent concerns about dual publication based on the content reviewed. However, it is important to confirm that the authors have adhered to ethical standards during their research and publication processes. Be transparent about any potential conflicts of interest and ensure all data sources are adequately cited.

We have added details on the authors adherence to ethical guidelines in the “Ethical Consideration” section.

• Formatting and Writing Style:
o There are minor grammatical issues and inconsistencies in formatting that should be addressed to improve the overall professionalism of the manuscript. A thorough proofreading session is recommended before resubmission.

We have addressed these gaps.

**Responses to reviewer 2**

Barring minor correction, the article is well written. It is crisp and concise. It identifies the gaps in the program well. The program requires strengthening at various levels to achieve the goal of elimination of mother to child transmission of HIV.

Thank you! We have started addressing these gaps to strengthen eMTCT.
